# cTfh Cell Activation and Changes in BTLA and LAG‐3 Expression Differentially Associate With Poor Pregnancy Outcome in *Plasmodium falciparum* Placental Malaria

**DOI:** 10.1155/jimr/6467951

**Published:** 2026-04-15

**Authors:** Bernard Marie Zambo Bitye, Chris Marco Mbianda Nana, Bodin Darcisse Kwanou Tchakounte, Balotin Fogang, Berenice Kenfack Tekougang Zangue, Reine Seumko’o Medouen Ndeumou, Lawrence Ayong, Rosette Megnekou

**Affiliations:** ^1^ Department of Animal Biology and Physiology, Faculty of Sciences, University of Yaoundé I, PO.BOX: 812, Yaoundé, Cameroon, uy1.uninet.cm; ^2^ Immunology Laboratory of the Biotechnology Center, University of Yaoundé I, PO.BOX: 17673, Etectak, Yaoundé, Cameroon, uy1.uninet.cm; ^3^ Malaria Research Unit, Centre Pasteur du Cameroun, PO.BOX: 1274, Yaoundé, Cameroon, pasteur-yaounde.org

**Keywords:** cTfh cells, immunoregulatory markers, placental malaria, pregnancy outcomes

## Abstract

Continuous exposure to antigens drives the activation of circulating T follicular helper (cTfh) cells, promoting high‐affinity antibody production. However, sustained stimulation may impair their function through the upregulation of immunoregulatory markers like B and T lymphocyte attenuator (BTLA) and *Lymphocyte-activation gene 3 (LAG-3)*. This study investigated the role of cTfh cells and their subsets in *Plasmodium falciparum*‐associated placental malaria (PM). In a cross‐sectional study conducted in Yaounde, Cameroon (March 2022–May 2023), peripheral, placental, and cord blood samples were collected from 49 women at delivery. Hemoglobin (Hb) levels were recorded, and peripheral blood mononuclear cells (PBMCs), intervillous blood mononuclear cell (IVBMCs), and cord blood mononuclear cells (CBMCs) were isolated for multiparametric flow cytometry analysis of cTfh cells and subsets (ICOS^−^ and ICOS^+^ cTfh subsets). PM was associated with increased cTfh cell frequencies in both PBMC and IVBMC. PM‐negative (PM−) women displayed higher ICOS^−^ cTfh cell frequencies, while PM‐positive (PM+) women had elevated ICOS^+^ cTfh cells that correlated with parasitemia levels. BTLA expression was downregulated in bulk cTfh and ICOS^−^ cTfh cells during PM. Interestingly, cTfh frequencies in IVBMC positively correlated with baby weight, while cTfh frequencies in PBMC and IVBMC negatively associated with maternal Hb. Furthermore, BTLA and LAG‐3 expression in IVBMC negatively correlated with birth weight. Together, these findings highlight altered cTfh cell profiles during PM, influencing maternal and neonatal outcomes.

## 1. Introduction

Placental malaria (PM) is associated with serious complications in pregnant women, their fetuses, and newborns. According to the WHO 2023 world malaria report, ~12.7 million women in the WHO African region were exposed to malaria infection during pregnancy, resulting in diverse health outcomes for both the pregnant mothers and their babies [1]. The increased rate of poor pregnancy outcomes such as maternal anemia, preterm delivery, and low‐birth‐weight babies [2, 3], results from the sequestration of *Plasmodium falciparum*‐infected red blood cells in placental tissues, leading to PM. *Plasmodium falciparum* sequestration is mediated by VAR2CSA antigens expressed on the surface of infected red blood cells, which bind to placental syncythiothrophoblast via Chondroitin Sulfate A (CSA) receptors, resulting in impaired placental function [4–6]. Malaria‐specific CD4^+^ T cells could play an important role in protection given their involvement in adaptive immune responses [7]. Placental infection by malaria parasites has been shown to lead to preimmunition characterized by the presence of anti‐VAR2CSA antibodies in peripheral blood [8]. However, the host’s ability to maintain an effective immune response remains not well understood.

The production of antibodies is orchestrated by CD4 T‐follicular helper (Tfh) cells, which express the CXCR5 chemokine receptor and play a key role in activating naïve B cells and their differentiation into antibody‐producing and memory B cells in germinal centers [9]. The circulating counterpart (circulating Tfh [cTfh]) of bona fide germinal center Tfh cells has recently been described [9]. Although cTfh cells share functional properties and express surface markers such as CXCR5, PD‐1, and ICOS, similar to those of germinal center Tfh cells, they lack the transcription factor BCL‐6 [10–12]. Strong T cell antigen receptor (TCR) signaling and continuous antigenic stimulation promote ICOS expression, a key costimulatory molecule on cTfh cells, which can adopt either an activated or resting phenotype [11, 13]. Activation of cTfh cells, which are characterized by expression of ICOS, leads to the production of IL‐2 and IL‐10 cytokines involved in B cell expansion and differentiation [12]. cTfh cells can be used as a less‐invasive and more accessible cell population to study the role of Tfh cells in pathogen‐associated immune responses.

Pregnancy alters maternal humoral immunity favoring antibody production by activated memory B cells [14–16]. Blood‐stage infection with *P. falciparum* has been associated with cTfh cell expansion and activation, contributing to the production of functional antibodies [17, 18]. Women in the third trimester of pregnancy, when compared to nonpregnant counterparts have higher proportions of functional cTfh cells [19, 20]. Chronic/repeated antigenic stimulation of cTfh results in increased surface expression of immunoregulatory markers such as cytotoxic T lymphocyte‐associated protein 4 (CTLA‐4), B and T lymphocyte attenuator (BTLA/CD272), *lymphocyte-activation gene 3 (LAG-3)*, leading to the impairment of its function [21]. Indeed, it has been shown that exhaustion markers CTLA‐4, BTLA/CD272, and LAG‐3 play a key role in immune tolerance by modulating TCR signaling [21]. BTLA, a member of the immunoglobulin superfamily, is expressed on B and T cells, macrophages, dendritic cells, and natural killer cells [22, 23]. It reduces T cell proliferation by binding to the herpesvirus‐entry mediator (HVEM) found on hematopoietic cells [24]. BTLA is upregulated in Tfh cells compared to naïve CD4^+^ T cells and negatively regulates Tfh support of GC B cells by restricting IL‐21 production [25, 26]. LAG‐3, a CD4‐related transmembrane protein that binds to MHC class II molecules, has been proposed to suppress T cell activation by competitively inhibiting CD4 engagement [27]. It has been shown that LAG‐3 expression in peripheral blood mononuclear cells (PBMCs) of malaria‐infected patients correlates with T cell activation and course of disease [28, 29].

While upregulation of exhaustion markers during acute infections may not directly indicate impaired cell function, it can reflect immune activation aimed at moderating excessive response [30]. However, studies of T cells in malaria have shown that the expression of coinhibitory receptors has a dichotomous function in limiting immune responses and regulating pathogen control [31–33]. These findings suggest that inhibitory pathways may provide an explanation for the lack of long‐term sterilizing immunity in malaria‐infected patients [34]. The role of cTfh cells and the expression of exhaustion markers such as BTLA and LAG‐3 on these cells in the context of *P. falciparum* PM remains poorly understood. This study aimed to characterize the cell profile and activation status of cTfh cells and the expression of immunoregulatory markers on these cells in relation to *Plasmodium falciparum* PM. Understanding the role of cTfh cells in PM may provide insights for the development of novel control tools against pregnancy‐associated malaria.

## 2. Materials and Methods

### 2.1. Ethics Statement

All procedures were approved by the National Ethics Committee of Cameroon (N°2021/02/1331/CE/CNERSH/SP). Administrative authorizations were obtained from the Cameroon Ministry of Public Health (N°631‐1221 of April 28, 2021, D30‐459/L/MINSANTE/SG/DROS) and the head of each health facility. Prior to enrollment into the study, written informed consent was obtained from each participant or legal guardian according to the Declaration of Helsinki. Upon enrollment, malaria rapid diagnostic test (RDT) was performed for each participant, and positive results were confirmed through thick blood or tissue smear microscopy. All positive results were promptly communicated to the attending physician for routine care as per national policy.

### 2.2. Study Population

Samples for this cross‐sectional study were collected from pregnant women at delivery between March 2022 and May 2023 at the Marie Reine Health Center of Etoudi and at the Catholic Hospital of “Marie des Anges” of Nkoabang, Yaounde, Cameroon. These two healthcare centers are located in two outlying districts of Yaounde, where malaria transmission is perennial, with two wet and two dry seasons.

Exclusion criteria were preexisting health conditions such as preeclampsia, diabetes, toxoplasmosis, hepatitis, syphilis, and HIV in pregnant women at enrollment.

### 2.3. Clinical Parameters and Sample Collection

Information on the participant’s age, gravidity, parity, gestational age, use of intermittent preventive treatment with sulfadoxine‐pyrimethamine during pregnancy (IPTp‐SP), use of long‐lasting insecticide‐treated bed nets (LLITN), and baby’s weight at delivery was recorded in a standard questionnaire. With respect to IPTp‐SP, mothers were asked to report the total number of doses received during pregnancy, and this information was cross‐checked with antenatal healthcare records when available. Birth weights lower than 2.5 kg were considered low as per the World Health Organization’s guidelines [35]. Maternal venous, intervillous, and cord blood samples were aseptically collected immediately after delivery from all selected participants in BD heparinized Vacutainer tubes (BEC 367880). A total of 49 pregnant women were included in this study, from whom peripheral, intervillous, and cord blood were collected. Additionally, placental tissue biopsies were collected from the maternal side of the placenta immediately following delivery and used to diagnose PM. All blood samples were additionally tested for *Plasmodium falciparum* infection using the One Step HRP‐II RDT and pLDH RDT (SD Bioline malaria antigen *P.f/*Pan, Standard Diagnostics Inc., Kyonggi‐do, Korea) to assess peripheral malaria infection and to guide the clinical management of malaria [36]; however, RDT results were not used for PM classification.

### 2.4. Diagnosis of PM and Determination of Hemoglobin (Hb) Levels

PM status was determined using microscopic examination of placental impression smears, which is considered the reference method of classification [37, 38]. Briefly, thick blood smears from maternal peripheral, placenta intervillous space, and cord blood as well as impression smears from placental tissue samples were stained using Giemsa and examined by at least two skilled microscopists for the presence of malaria parasites. Parasitemia in the various preparations was determined according to WHO recommendations and expressed as parasites per microliter of blood in the case of thick blood smears and as percent infected red blood cells in the case of placental tissue impression smears. Participants were considered PM positive (PM+) when infected erythrocytes were detected in placental impression smears of placenta tissue and as PM negative (PM−) when no parasites were observed in these smears. Hb levels in maternal and neonate cord blood were determined using a hemoglobinometer (ACON Laboratories, INC, San Diego, USA). Participants were considered anemic if Hb < 11 g/dL, whereas neonates were considered anemic if Hb < 12.5 g/dL [39].

### 2.5. PBMCs, Intervillous Blood Mononuclear Cells (IVBMCs), and Cord Blood Mononuclear Cells (CBMCs) Isolation

Fresh PBMCs, IVBMCs from the mother and intervillous space of the placenta, respectively, as well as CBMCs from the umbilical cord, were isolated as previously described [40]. Briefly, peripheral, intervillous, or cord blood samples were individually diluted 1:1 in PBS 1X (without Ca^2+^ and Mg^2+^) supplemented with 2% FBS (Thermo Fisher Scientific, USA). The diluted samples were layered over 15 mL of Histopaque 1077 (Sigma–Aldrich, USA) in a 50 mL Falcon tube. The tubes were centrifuged for 30 min at 800 g and 20°C. Different layers of isolated PBMCs, IVBMCs, or CBMCs were collected, washed with PBS 1X (without Ca^2+^ and Mg^2+^), supplemented with 2% FBS, centrifuged at 600 g and 20°C for 8 min, and the pellet resuspended in 1xPBS. Trypan blue was used to assess the viability of the isolated cells. Only samples with cell viability greater than 95% were used for surface staining. For surface staining, around were resuspended in 1 mL of 1X PBS.

### 2.6. Surface Staining and Flow Cytometry

Approximately 1 × 10^6^ isolated PBMCs, IVBMCs, or CBMCs cells were transferred in each well of a 96‐well V‐shaped bottom microplate, washed by centrifugation, and resuspended in 50 µl of FACS buffer (1X PBS without Ca^2+^ and Mg^2+^, 5% FBS, 5 mM EDTA). The cells were then incubated in the dark with human FcR blocking reagent (Miltenyi Biotech) for 10 min at 4°C. The cells were further washed, stained with 50 µl of an appropriate combination of fluorescently labeled monoclonal antibodies for 30 min at 4°C in the dark. Cells were then washed with 200 µl of FACS buffer at 1800 rpm for 3 min and resuspended in FACS buffer prior to acquisition. Data were acquired using BD FACS CANTO II and analyzed using FlowJo software version 10.8.1 (Tree Star, Inc.).

The following monoclonal antibodies were used for the staining: brilliant violet (BV) 510 labeled anti‐CD3 (clone UCHT1) (BD Biosciences, USA); phycoerythrin (PE) labeled anti‐CD4 (clone SK3) (BD Biosciences), USA); BV421 labeled anti‐CXCR5 (CD185; clone RF8B2; BD Biosciences, USA); PE labeled anti‐CD278 (ICOS; clone DX29) (BD Biosciences); fluorescein isothiocyanate (FITC) labeled anti‐CD223 (LAG‐3; clone 3DS223H; eBioScience/ThermoFisher Scientific); allophycocyanin (APC) labeled anti‐CD279 (PD‐1; clone EH12.2H7; Biolegend); and APC‐cyanine 7 (APC‐Cy7) labeled anti‐CD272 (BTLA; clone MIH26; Biolegend).

### 2.7. Statistical Analysis

All statistical analysis were performed using GraphPad Prism version 8.4.3. Results are generally presented as means with standard deviation or medians with interquartile ranges unless otherwise specified. Mann–Whitney rank sum test was used for comparisons between two groups, while the Spearman rank order correlation coefficient (*r*_s_) was used to evaluate associations. Proportions were compared using Fisher’s exact test. A significance level of 95% was used.

## 3. Results

### 3.1. Study Population

A total of 49 women in labor participated in this study, of whom 28 were PM− and 21 were PM+. The general characteristics of the study population are summarized in Table 1. No significant difference was observed between PM− and PM+ regarding mean age, median gestational age, parity, and gravidity (*p* = 0.225; *p* = 0.24; *p* = 0.061; *p* = 0.43, respectively). Maternal Hb levels were significantly lower (*p* = 0.003) in PM+ women (median: 10.67 g/dL) compared to PM− women (median: 12.66 g/dL). Furthermore, the proportion of participants with anemia was significantly higher (*p* = 0.003) in PM+ women (78.57%) compared to PM− women (21.43%). No significant difference was observed in fetal Hb levels or the proportion of fetal anemia cases between PM+ (median: 15.66 g/dL and 38.10%, respectively) and PM− women (14.66 g/dL and 61.9%, respectively) (*p* = 0.244 and *p* = 0.771). Baby birth weight at delivery was significantly lower in PM+ women (mean: 3045 g) compared to PM− women (mean: 3355 g) (*p* = 0.015). No significant difference in the proportion of women who took IPTp‐SP and used ITNs during pregnancy (*p* = 0.999 and *p* = 0.683) between PM+ (43.9% and 45.24%, respectively) and PM− (56.1% and 54.76%, respectively) women.

**Table 1:** General characteristics of the study population.

Parameters	PM− women (*n* = 28)	PM+ women (*n* = 21)	*p* value
Age in years (mean ± SD)	27.39 ± 5.138	25.62 ± 4,801	0.225
Gestational age, median [25%–75% IQR]	39 [38–41]	39 [37.25–40]	0.24
Parity [median and 25%–75% IQR]	2.5 [1.25–4]	2 [1 – 2.5]	0.061
Gravidity [median and 25%–75% IQR]	3 [2–4]	2 [1–4]	0.430
RDT positives (%)	4 (14.3)	21 (100.0)	—
Peripheral thick blood smear microscopy positives (%)	0 (0.0)	15 (71.4)	—
Placenta intervillous thick blood smear microscopy positives (%)	0 (0.0)	13 (61.9)	—
Cord thick blood smear microscopy positives (%)	0 (0.0)	0 (0.0)	—
Maternal hemoglobin levels in g/dL, median [25%–75% IQR]	12.66 [11.66–14]	10.67 [10.33 – 12.33]	**0.003**
Percentage of maternal anemia (%)	3/27 (21.43)	11/21 (78.57)	**0.003**
Fetal hemoglobin levels in g/dL, median [25%–75% IQR]	14.66 [13–16]	15.66 [13.84 – 16.50]	0.244
Percentage of fetal anemia (%)	13/28 (61.90)	8/21 (38.10)	0.771
Baby birth weight (mean g ± SD)	3355 ± 405.3	3045 ± 413.1	**0.015**
IPTp‐SP use during pregnancy (≥1 dose) (%)	23/26 (56.10)	18/21 (43.90)	>0.999
ITNs usage (%)	23/27 (54.76)	19/21 (45.24)	0.683

*Note:* Data were obtained by maternal self‐report and confirmed using antenatal healthcare booklets. In the absence of such a booklet, the participant was not included in calculations relating to IPTp‐SP use. %, percentage; PM+, placental malaria positive women; PM−, placental malaria negative women. Bold values show the significance.

Abbreviations: IPTp‐SP, intermittent preventive treatment with sulphadoxine‐pyrimethamine; IQR, interquartile ranges; ITNs, insecticide treated bed net; SD, standard deviation.

### 3.2. Dynamics of Activated cTfh Cell Phenotypes in PBMC, IVBMC, and CBMC in Women at Delivery

**Figure 1:**
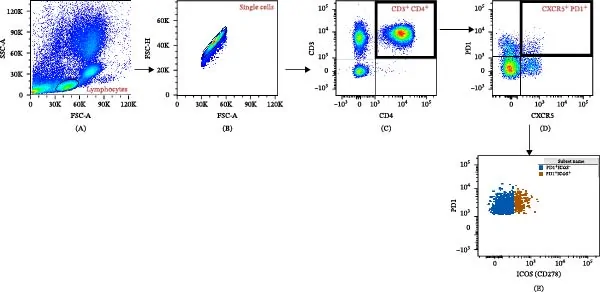
Gating strategy of circulating T follicular helper cells. Samples were initially gated on (A) total lymphocytes based on SSC‐A/FSC‐A gating, followed by (B) single cells gating using FSC‐H/FSC‐A, and (C) CD4 T cell gating from singlets as CD3^+^CD4^+^ cells. (D) Circulating T follicular helper (cTfh) cell subsets were gated from CD3^+^CD4^+^ as CXCR5^+^PD1^+^, whereas (E) activated and resting cTfh were characterized using ICOS as ICOS^+^cTfh and ICOS^−^cTfh, respectively. Data are derived from a single experiment using independent biological samples.

We investigated the dynamics of cTfh cells and its subsets in different blood compartments through multiparametric flow cytometry according to the gating strategy shown in Figure 1. As reported previously, cTfh cells were defined as CD3^+^CD4^+^CXCR5^+^PD‐1^+^ [41]. The results show that the frequency of cTfh cells among CD3^+^CD4^+^ cells was significantly higher in PBMC and IVBMC compared to CBMC (*p*  < 0.0001 for both). No significant difference was observed between the frequency of cTfh cells in PBMC and IVBMC (*p* = 0.125) (Figure 2A).

Figure 2Frequencies of cTfh cells and subsets across blood compartments in women at delivery. cTfh cell frequencies were analyzed through multiparametric flow cytometry in PBMC, IVBMC and CBMC of women at delivery and presented independently of placental malaria infection status. (A) Frequency of total cTfh cells, (B) frequency of ICOS^−^cTfh cells, (C) frequency of ICOS^+^cTfh cells, and D) comparison between frequencies of ICOS^−^cTfh and ICOS^+^cTfh cells in each compartment. Frequencies were compared using Mann–Whitney test. Significant differences correspond to *p* value  < 0.05. Sample size: PBMC (*N* = 49); IVBMC (*N* = 45), and CBMC (*N* = 48). Data are derived from a single experiment using independent biological samples. CBMC, cord blood mononuclear cell; IVBMC, intervillous blood mononuclear cell; PBMC, peripheral blood mononuclear cell. Each dot represents a single individual.

**Figure:**
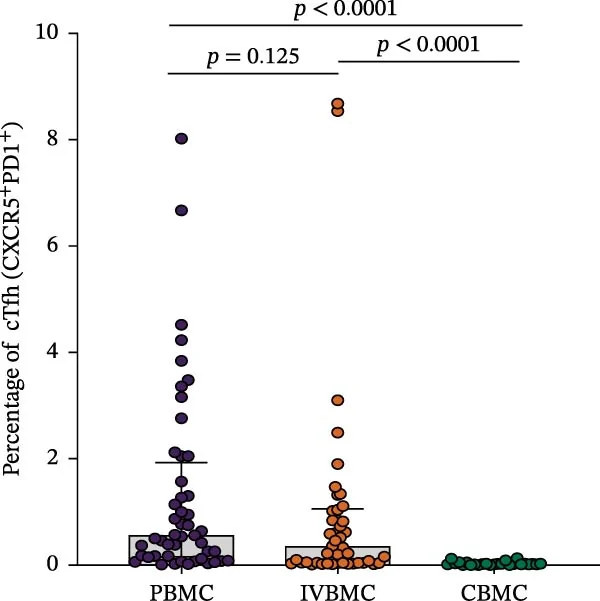
(A)

**Figure:**
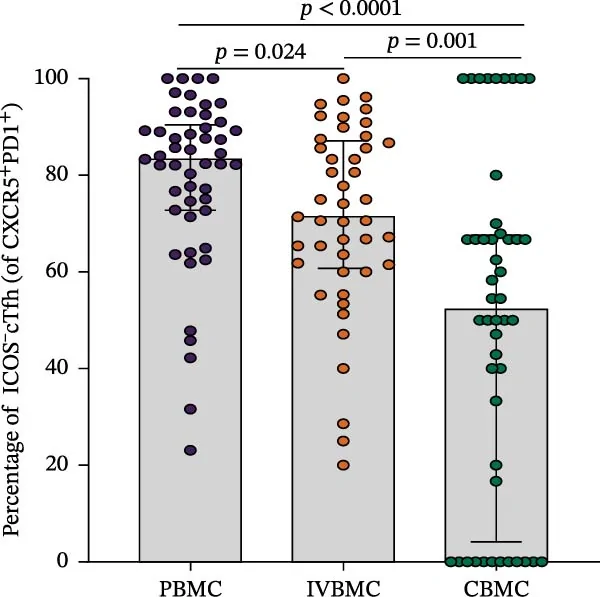
(B)

**Figure:**
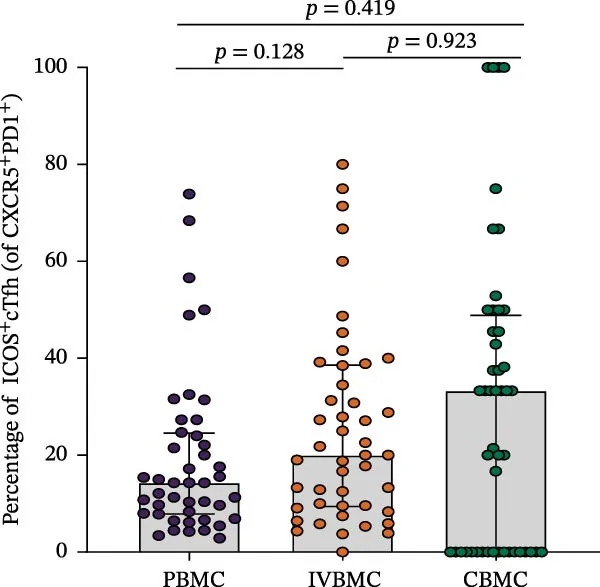
(C)

**Figure:**
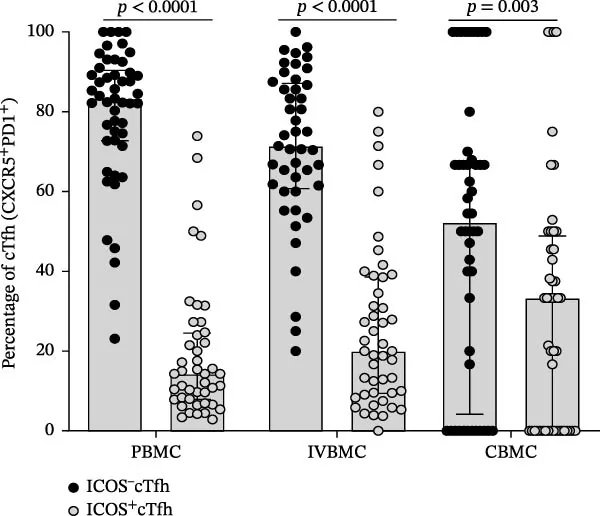
(D)

Regarding the activation status of cTfh, resting and activated cTfh cells were defined as the nonexpression or expression of surface marker ICOS (ICOS^−^cTfh cells and ICOS^+^cTfh, respectively) [42, 43]. The results show that the frequency of ICOS^−^ cTfh cells were significantly higher in PBMC and IVBMC compared to CBMC (*p*  < 0.0001 and *p* = 0.001). Similarly, the frequency of ICOS^−^cTfh cells was significantly higher in PBMC compared to IVBMC (*p* = 0.024; Figure 2B). In contrast, although the frequencies of ICOS^+^cTfh cells were higher in CBMC and IVBMC than in PBMC, these differences were not significant (Figure 2C).

In each blood compartment, ICOS^−^cTfh cells predominated over the ICOS^+^ cTfh cell population (<0.0001 ≤ *p* ≤ 0.003) (Figure 2D). Together, these results suggest that in women at delivery, the frequency of cTfh and its subsets varies across different blood compartments.

### 3.3. Association of *P. falciparum* PM With Decreased Frequency of Resting cTfh and Increased Frequency of Activated cTfh in PBMC and IVBMC but Not in CBMC

**Figure 4:**
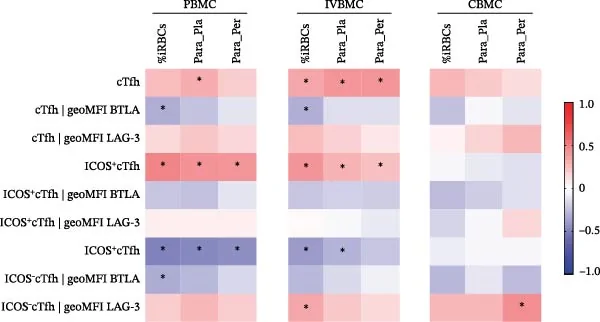
Heatmap depicting the correlation between the frequency of cTfh cell phenotypes and parasitemia. The correlation between cTfh cells in PBMC, IVBMC, CBMC, and parasitemia was determined using Spearman’s rank order correlation test. Significant differences correspond to *p* value  < 0.05. Data are derived from a single experiment using independent biological samples. Sample size: PBMC (*N* = 49); IVBMC (*N* = 45), and CBMC (*N* = 48). %iRBC: Percent infected red blood cell based on placenta tissue impression smear; Para_Per: peripheral blood parasitemia; Para_Pla: placental blood parasitemia. CBMC, cord blood mononuclear cell; IVBMC, intervillous blood mononuclear cell; PBMC, peripheral blood mononuclear cell.  ^∗^indicates the significance of *p* value.

Generally, the frequency of cTfh cells in PBMC and IVBMC was higher in PM+ women compared to PM− women, although not significant (*p* = 0.151, *p* = 0.091, respectively) (Figure 3A). The frequency of cTfh in PBMC correlated positively with placental blood parasitemia (*r*_s_ = 0.314, *p* = 0.030) (Figure 4 and Table 2A). Similarly, the frequency of cTfh in IVBMC correlated positively with parasitemia from placenta tissue impression smear, placental blood, and peripheral blood (0.339 ≤ *r*_s_ ≤ 0.417; 0.004 ≤ *p* ≤ 0.021; Figure 4 and Table 2B), suggesting association of cTfh cell frequency with *P. falciparum* parasitemia.

Figure 3Frequencies of total cTfh cells and subsets in PM+ women compared to PM− women at delivery. cTfh cells and subsets were analyzed through multiparametric flow cytometry in PBMC, IVBMC and CBMC of PM− (in blue) and PM+ (in red) women. (A) Frequency of total cTfh cells, (B) frequency of ICOS^−^cTfh cells, and (C) frequency of ICOS^+^cTfh cells in each blood compartment. Frequencies were compared using Mann–Whitney test. Significant differences correspond to *p* value  < 0.05. Data are derived from a single experiment using independent biological samples. Sample size: PBMC (PM+, *N* = 21; PM−, *N* = 28); IVBMC (PM+, *N* = 21; PM−, *N* = 24); CBMC (PM+, *N* = 20; PM−, *N* = 28). CBMC, cord blood mononuclear cell; IVBMC, intervillous blood mononuclear cell; PBMC, peripheral blood mononuclear cell; PM+, placental malaria positive women; PM−, placental malaria negative women. Each dot represents a single individual.

**Figure:**
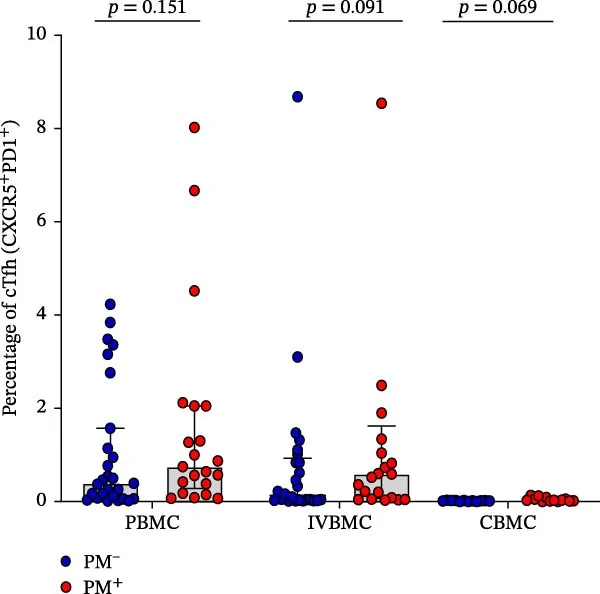
(A)

**Figure:**
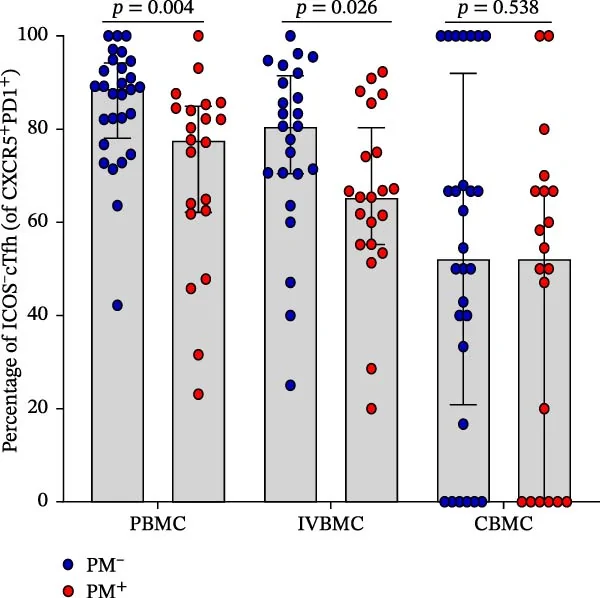
(B)

**Figure:**
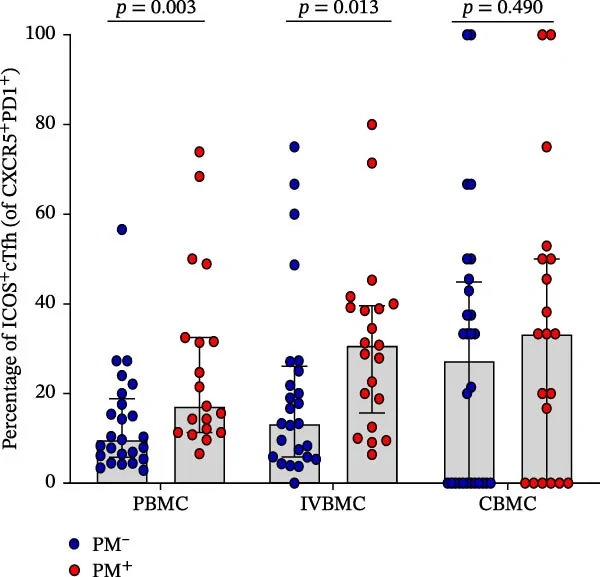
(C)

**Table 2:** Correlations between the frequency of cTfh cells in PBMC, IVBMC, and CBMC and parasitemia.

A					B					C				
PBMC		%iRBCs	Para_Pla	Para_Per	IVBMC		%iRBCs	Para_Pla	Para_Per	CBMC		%iRBCs	Para_Pla	Para_Per
**cTfh**	*r*_s_	0.253	**0.314**	0.193	**cTfh**	*r*_s_	**0.339**	**0.417**	**0.404**	**cTfh**	*r*_s_	0.28	0.208	0.137
	*p* value	0.083	**0.030**	0.198		*p* value	**0.021**	**0.004**	**0.006**		*p* value	0.175	0.319	0.514
**cTfh | geoMFI BTLA**	*r*_s_	**−0.320**	−0.232	−0.110	**cTfh | geoMFI BTLA**	*r*_s_	**−0.311**	−0.134	−0.135	**cTfh | geoMFI BTLA**	*r*_s_	−0.249	−0.046	−0.107
	*p* value	**0.034**	0.129	0.488		*p* value	**0.054**	0.416	0.425		*p* value	0.132	0.784	0.523
**cTfh | geoMFI LAG-3**	*r*_s_	0.146	0.219	0.153	**cTfh | geoMFI LAG-3**	*r*_s_	0.253	0.184	0.092	**cTfh | geoMFI LAG-3**	*r*_s_	0.052	0.168	0.277
	*p* value	0.322	0.134	0.311		*p* value	0.093	0.226	0.559		*p* value	0.748	0.299	0.084
**ICOS** ^**+**^ **cTfh**	*r*_s_	**0.479**	**0.417**	**0.407**	**ICOS** ^**+**^ **cTfh**	*r*_s_	**0.41**	**0.293**	0.244	**ICOS** ^**+**^ **cTfh**	*r*_s_	0.018	−0.091	−0.13
	*p* value	**0.001**	**0.005**	**0.007**		*p* value	**0.005**	**0.048**	0.111		*p* value	0.905	0.539	0.38
**ICOS** ^**+**^ **cTfh | geoMFI BTLA**	*r*_s_	−0.221	−0.248	−0.110	**ICOS** ^**+**^ **cTfh | geoMFI BTLA**	*r*_s_	−0.228	−0.189	−0.201	**ICOS** ^**+**^ **cTfh | geoMFI BTLA**	*r*_s_	−0.259	−0.201	−0.124
	*p* value	0.177	0.128	0.510		*p* value	0.158	0.243	0.226		*p* value	0.174	0.296	0.522
**ICOS** ^**+**^ **cTfh | geoMFI LAG-3**	*r*_s_	0.060	0.065	0.048	**ICOS** ^**+**^ **cTfh | geoMFI LAG-3**	*r*_s_	0.023	−0.039	−0.091	**ICOS** ^**+**^ **cTfh | geoMFI LAG-3**	*r*_s_	−0.178	−0.008	0.155
	*p* value	0.704	0.681	0.762		*p* value	0.884	0.804	0.566		*p* value	0.348	0.965	0.413
**ICOS** ^−^ **cTfh**	*r*_s_	**−0.493**	**−0.468**	**−0.447**	**ICOS** ^−^ **cTfh**	*r*_s_	**−0.395**	**−0.298**	−0.226	**ICOS** ^−^ **cTfh**	*r*_s_	−0.077	−0.019	0.01
	*p* value	**<0.0001**	**0.001**	**0.002**		*p* value	**0.007**	**0.047**	0.145		*p* value	0.602	0.896	0.947
**ICOS** ^−^ **cTfh | geoMFI BTLA**	*r*_s_	**−0.322**	−0.281	−0.154	**ICOS** ^−^ **cTfh | geoMFI BTLA**	*r*_s_	−0.276	−0.149	−0.064	**ICOS** ^−^ **cTfh | geoMFI BTLA**	*r*_s_	−0.274	−0.105	−0.26
	*p* value	**0.035**	0.068	0.335		*p* value	0.094	0.371	0.711		*p* value	0.122	0.561	0.143
**ICOS** ^−^ **cTfh | geoMFI LAG-3**	*r*_s_	0.198	0.271	0.192	**ICOS** ^−^ **cTfh | geoMFI LAG-3**	*r*_s_	**0.339**	0.211	0.147	**ICOS** ^−^ **cTfh | geoMFI LAG-3**	*r*_s_	0.284	0.277	**0.439**
	*p* value	0.177	0.062	0.200		*p* value	**0.024**	0.169	0.352		*p* value	0.098	0.107	**0.008**

*Note:* %iRBC, percent infected red blood cell based on placenta tissue impression smear; Para_Per, peripheral blood parasitemia; Para_Pla, placental blood parasitemia. The bold values indicate the significance of the *p*‐value.

Abbreviations: CBMC, cord blood mononuclear cell; IVBMC, intervillous blood mononuclear cell; PBMC, peripheral blood mononuclear cell.

Regarding cTfh cell activation, the frequencies of ICOS^−^cTfh in PBMC and IVBMC was significantly lower in PM+ compared to PM− women (*p* = 0.004 and *p* = 0.026, respectively) while the frequencies of ICOS^+^cTfh was higher in PM+ compared to PM− women (*p* = 0.003 and *p* = 0.003, respectively) (Figure 3B,C). Furthermore, parasitemia correlated negatively with the frequency of ICOS^−^cTfh cells in PBMC and IVBMC (−0.493 ≤ *r*_s_ ≤ −0.298; 0.0001 ≤ *p* ≤ 0.047) and positively with that of ICOS^+^cTfh cells in PBMC and IVBMC (0.293 ≤ *r*_s_ ≤ 0.479; 0.001 ≤ *p* ≤ 0.048) (Figure 4; Table 2A,B). No association was observed between cTfh cell frequencies in CBMCs and PM (Table 2C). These findings suggest that interactions between malaria parasites and cTfh cells in PBMCs and IVBMCs may lead to differentiation into activated (ICOS^+^) cTfh, which may be involved in the control of malaria parasitemia.

### 3.4. Downregulation of Inhibitory BTLA Receptor Expression in Resting cTfh Cells During *P. falciparum* PM

T cell activation is generally associated with increased expression of immunoregulatory markers. In this study, we investigated the association between the expression levels of BTLA and LAG‐3 immune checkpoints in cTfh, ICOS^−^cTfh, and ICOS^+^cTfh cells from different blood compartments and *P. falciparum* placental infection. BTLA expression in both total cTfh and ICOS^−^cTfh was significantly lower in PM+ compared to PM− women in PBMC and IVBMC (*p* = 0.007 and *p* = 0.023 for cTfh, and *p* = 0.010 and *p* = 0.048 for ICOS^−^cTfh, respectively; Figure 5A,B). In addition, BTLA expression in PBMC correlated negatively with parasitemia from placenta tissue impression smear (*r*_s_ = −0.320; *p* = 0.034), and presented a borderline negative correlation in IVBMC (*r*_s_ = −0.311; *p* = 0.054; Figure 4 and Table 2A,B). In CBMC, BTLA expression in total cTfh and ICOS^−^cTfh was also lower in PM+ compared to PM− women, albeit not significant (*p* = 0.151 for cTfh and *p* = 0.178 for ICOS^−^cTfh). BTLA expression in activated cTfh (ICOS^+^cTfh) cells in all three compartments was also lower in PM+ compared to PM− women, although not significant (*p*  > 0.05). On the other hand, LAG‐3 expression in cTfh cells and ICOS^−^cTfh cells from all three compartments tends to be higher in PM+ compared to PM− women although only significant for ICOS^−^cTfh in IVBMC (*p* = 0.024; Figure 5D,E). In addition, LAG‐3 expression in ICOS^−^cTfh cells of IVBMC correlated positively with placenta tissue impression smear parasitemia (*r*_s_ = 0.339, *p* = 0.024) (Figure 4 and Table 2B). LAG‐3 expression in activated cTfh (ICOS^+^cTfh) cells in all three compartments was lower in PM+ compared to PM− women, although not significant (*p*  > 0.05). Together, these results suggest that placental infection with *P. falciparum* may interfere with the activity predominantly of resting cTfh cells in IVBMC.

Figure 5Expression levels of BTLA and LAG‐3 within cTfh cells in women at delivery. Mean fluorescence intensity (MFI) of BTLA and LAG‐3 within cTfh cells and subsets was analyzed through multiparametric flow cytometry in PBMC, IVBMC, and CBMC of PM− (in blue) and PM+ (in red) women. The figure shows the geometric mean fluorescence intensity of BTLA within cTfh cells (A), ICOS^−^cTfh cells (B), and ICOS^+^cTfh cells (C), and mean fluorescence intensity of LAG‐3 within cTfh cells (D), ICOS^−^cTfh cells (E), and ICOS^+^cTfh cells (F). Data are derived from a single experiment using independent biological samples. Sample size: PBMC (PM+, *N* = 21; PM−, *N* = 28); IVBMC (PM+, *N* = 21, PM−, *N* = 24); CBMC (PM+, *N* = 20; PM−, *N* = 28). Mean fluorescence intensities were compared using Mann–Whitney test. Significant differences correspond to *p* value  < 0.05. CBMC, cord blood mononuclear cell; IVBMC, intervillous blood mononuclear cell; PBMC, peripheral blood mononuclear cell; PM+, placental malaria positive women; PM−, placental malaria negative women. Each dot represents a single individual.

**Figure:**
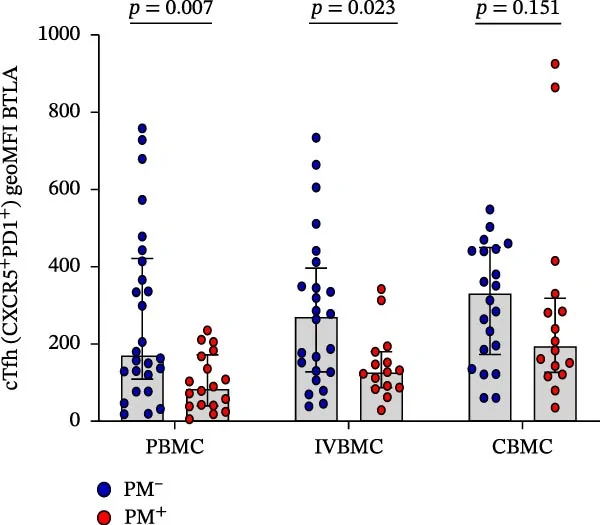
(A)

**Figure:**
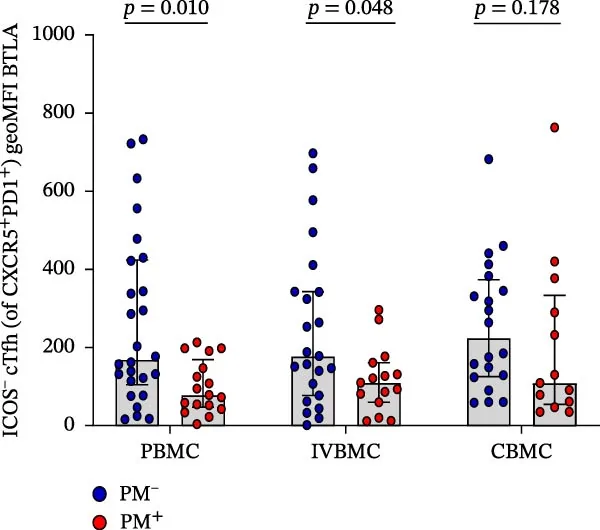
(B)

**Figure:**
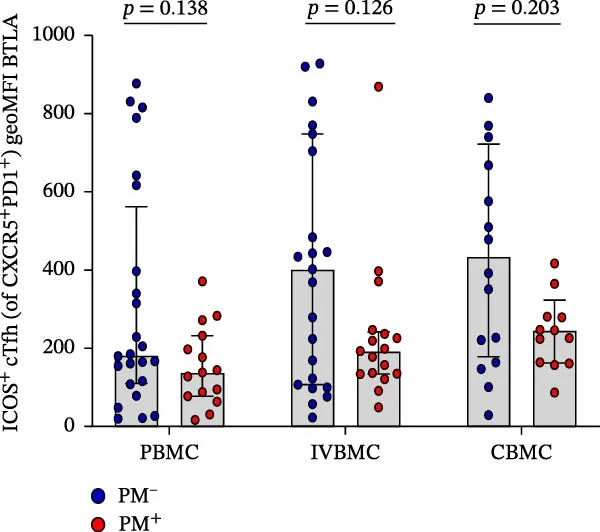
(C)

**Figure:**
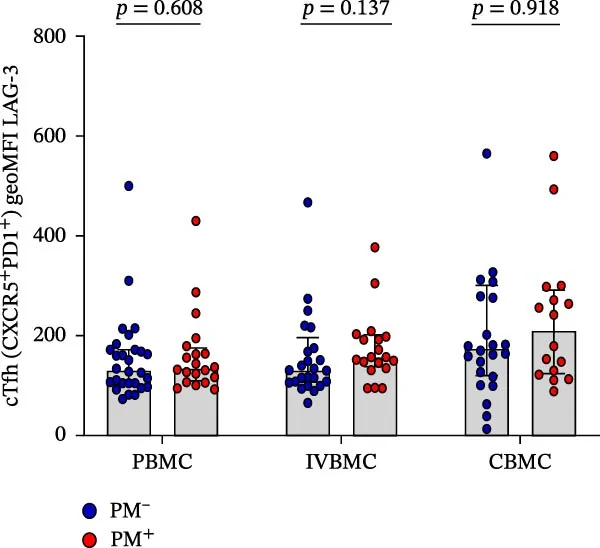
(D)

**Figure:**
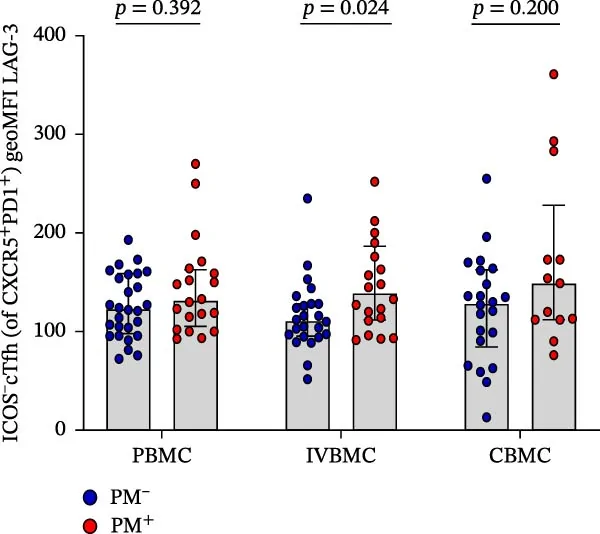
(E)

**Figure:**
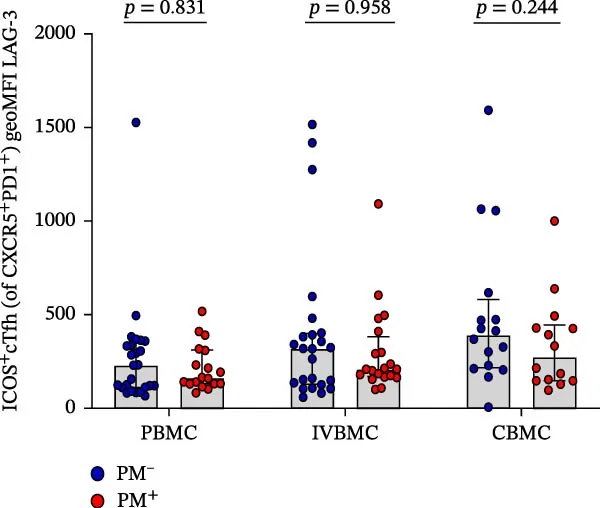
(F)

### 3.5. Association of cTfh Cell Activation and Increased Expression of Exhaustion Markers With Poor Pregnancy Outcome

**Figure 6:**
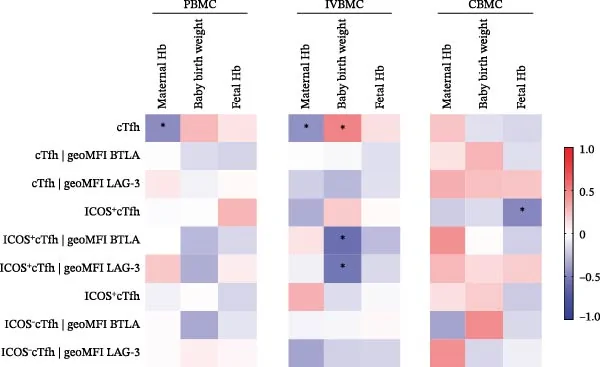
Heatmap depicting the correlation between the frequency of cTfh cells and pregnancy outcomes. The correlation between the frequency of cTfh cells in PBMC, IVBMC, CBMC and pregnancy outcome was determined using Spearman’s rank‐order correlation test. Significant differences correspond to *p* value  < 0.05. Data are derived from a single experiment using independent biological samples. Sample size: PBMC (*N* = 49); IVBMC (*N* = 45), and CBMC (*N* = 48). CBMC, cord blood mononuclear cells; Hb, hemoglobin level; IVBMC, intervillous blood mononuclear cell; PBMC: Peripheral blood mononuclear cell.  ^∗^indicates the significance of *p* value.

Phenotypic changes in T cells can be associated with pregnancy outcomes (protection or severity). As shown in Figure 6 and Supporting Information 1: Table S1, maternal Hb levels in PBMC and IVBMC correlated negatively with the frequency of cTfh cells (*r*_s_ = −0.454, *p* = 0.039 and *r*_s_ = −0.439, *p* = 0.046, respectively for PBMC and IVBMC]. In CBMC, fetal Hb levels also correlated negatively with the frequency of ICOS^+^cTfh cells (*r*_s_ = −0.471; *p* = 0.036). With the exception of the frequency of ICOS^+^cTfh cells in PBMC, which correlated negatively with maternal age (*r*_s_ = −0.369, *p* = 0.014) and parity (*r*_s_ = −0.332, *p* = 0.028), cTfh cell activation generally did not associate with maternal age or parity (Supporting Information 2: Table S2A–C). Similarly, BTLA and LAG‐3 expression within ICOS^+^cTfh cells correlated negatively with baby birth weight (*r*_s_ = −0.562, *p* = 0.031 and *r*_s_ = −0.537, *p* = 0.022, respectively, Figure 6 and Supporting Information 1: Table S1), suggesting an association between cTfh cell activity and poor pregnancy outcome.

## 4. Discussion

cTfh have emerged over the years as a major component of protective immunity, with evidence indicating their association with antibody induction during infections such as in malaria [44]. Although cTfh cells generally play a central role in modulating the adaptive immune response, its subsets have distinct signaling pathways [45]. In the context of *Plasmodium falciparum* PM infection in women at delivery, the phenotypic profile of cTfh cells and its subsets remain poorly understood. Closing this gap could provide insights into the potential involvement of cTfh cells in PM pathogenesis and may lead to new strategies for protection against PM. This study, therefore, aimed to characterize the cellular profiles, activation status, and the expression of immunoregulatory markers in cTfh cells in relation to *Plasmodium falciparum* PM.

The results show significant variations in frequencies of cTfh cells between blood compartments (PBMC, IVBMC, and CBMC). Indeed, cTfh cells were more frequent in PBMC and IVBMC than in CBMC. These findings are consistent with observations that pregnancy may enhance the humoral immune response by upregulating cTfh in PBMC during pregnancy [19]. Indeed, high estrogen levels during pregnancy are thought to increase the frequency of Tfh cells, enhancing humoral immunity and balancing Th1/Th2 responses to promote a successful pregnancy [19, 20]. In addition, it has been reported that Tfh cells have high frequencies in the placenta during late gestation [20]. Prior research on pregnancy‐associated malaria has shown notable changes in leukocyte and cytokine profiles at the maternal–fetal interface, including high numbers of leukocytes in placental versus peripheral blood, as well as increased placental inflammatory cells and pro‐inflammatory cytokines (IFN‐γ and TNF‐α) [46]. These changes in the cytokine milieu and immune cell composition have been linked to malaria pathophysiology and may affect the local immune environment of cTfh cells. However, the precise physiological role and mechanisms of Tfh cells in PM remain unclear. Current evidence suggests that Tfh cells can differentiate into cTfh cells, which diversify into subsets based on the expression of surface markers such as CCR6, CXCR3, PD‐1, CCR7, and ICOS.

ICOS is a functional marker of Tfh cells, which belongs to the CD28 family and plays an important role in Tfh cell recruitment to the follicle and its maintenance. In our study, the activation status of cTfh was classified as “resting” (ICOS^−^ cTfh cells) or “activated” (ICOS^+^ cTfh) cTfh cells [42, 43]. We compared these subsets across blood compartments and found that the frequency of ICOS^−^ cTfh was higher than ICOS^+^ cTfh in all blood compartments, consistent with previous studies [41, 47]. This suggests that ICOS^−^ cTfh cells may be essential for normal pregnancy. Indeed, recent studies link increased ICOS^+^ Tfh cells during pregnancy with heightened maternal B lymphocyte activation, potentially contributing to fetal rejection of the fetus in cases of recurrent spontaneous abortion [45]. Additionally, a high frequency of Tfh cells has been observed in preeclampsia women compared to healthy pregnant women [48].

Circulating Tfh cells may function through the expression of specific markers and/or secretion of various cytokines [18, 44, 49]. Our study reveals that placental *P. falciparum* infection affects cTfh cells and their subsets in each compartment, with consequences for both the pregnant women’s and fetal immunity. For the first time, we show a high frequency of cTfh cells in peripheral and placental blood of PM+ women compared to PM− women. These findings suggest that placental *P. falciparum* malaria infection stimulates Tfh cell proliferation and differentiation in these blood compartments, potentially contributing to the altered cytokine milieu observed in maternal, placental, and cord blood, which previously has been linked to maternal anemia and reduced birth weight [50–52]. Consistent with this, studies from Indonesia have shown robust cTfh cell activity during blood‐stage malaria in adults, leading to increased B‐cell responses and antibody induction [18].

Regarding cTfh cell activation during PM, we show for the first time that, peripheral and in intervillous placental blood, the magnitude of ICOS^+^ cTfh was higher in PM+ women than in PM− women and correlated positively with parasitemia. In contrast, ICOS^−^ cTfh cells were higher in PM− women than in PM+ women and increased with decreasing parasitaemia. This suggests that parasite presence in peripheral and placental blood induces cTfh cell differentiation into an activated cTfh cell subset. Consistent with our data, high frequencies of ICOS^+^ cTfh cells have been described in other infectious diseases [18, 53–56]. ICOS, a key secondary costimulatory molecule expressed on the surface of activated T cells [57], is essential for Tfh‐B cell interactions and regulates Tfh cell differentiation and GC formation through PI3K signaling [58, 59]. Rasheed et al. [60] reported that ICOS^+^ T cells in tonsils secrete high levels of IL‐21 and CXCL13, which are essential for B cell maturation and immunoglobulin production, suggesting that GC Tfh cells with high ICOS expression represent terminally differentiated subsets exhibiting efficient B helper activity [60].

T‐cell activation is generally associated with the expression of immune checkpoint markers. The exhaustion marker, BTLA, CD272 is crucial for maintaining T cell tolerance by interacting with its ligand, the HVEM [61]. Disruptions in the BTLA‐HVEM pathway have been associated with increased susceptibility to infections [62] as well as the development of autoimmune diseases [63]. In this study, BTLA expression was downregulated within bulk cTfh and ICOS^−^ cTfh cells in women with *P. falciparum* PM, suggesting that malaria parasites may disrupt resting cTfh cell activity. These results are consistent with findings in chronic lymphocytic leukemia, showing the dependence of BTLA levels on the activation state of T cells; their expression being low in naïve cells, increasing upon activation, and decreasing in fully activated cells [64, 65]. Furthermore, we show for the first time that LAG‐3 expression was associated with resting cell activity in IVBMC during PM. These findings contrast observations in healthy humans, indicating the absence of LAG‐3 expression in resting T‐cells [30]. Given that the placenta represents a unique immunological niche, it is plausible that chronic low‐level antigenic stimulation following *P. falciparum* infection may activate resting cTfh cells to express LAG‐3, essential for limiting immune‐mediated pathology at the maternal–fetal interface. Infection chronicity may also influence cTfh activation, with acute versus chronic exposure potentially shaping distinct activation and regulatory profiles [66, 67]. However, the low number of identified chronic cases (presence of malaria pigment in placental impression smears) in the study population precluded such analyses in this study.

cTfh cells have been shown to contribute to the pathophysiology of malaria in children and adults. Here, we reveal for the first time the association between cTfh cell phenotypes and pregnancy outcome. In fact, bulk cTfh cell expression in PBMC and IVBMC was negatively associated with materna Hb levels. Additionally, ICOS^+^ cTfh cells expressing BTLA and LAG‐3 in IVBMC were associated negatively with baby birth weight. In contrast, bulk cTfh cell expression in IVBMC was associated positively with baby weight. These observations suggest a beneficial role of cTfh cells on the fetus and a potentially harmful effect on the mother. Solders et al. [68] observed that decidual CD4+ T cells, expressing exhaustion markers, were upregulated in full‐term pregnancy [68], and such markers have been implicated in the pathophysiology of pregnancy loss [69–71].

Fetal anemia associated with PM is a risk factor for infant mortality. We show for the first time that the frequency of ICOS^+^ cTfh cells in CBMC is negatively associated with fetal Hb levels. Although the precise mechanism by which ICOS^+^ cTfh cells impact fetal Hb levels remains unclear, we speculate that this may be attributed to damaging inflammatory responses of activated cTfh cells resulting from placental infection by malaria parasites [72–74]. We show that maternal age and parity correlate positively with ICOS^−^cTfh cells and negatively with ICOS^+^cTfh cell frequencies in PBMC. These results suggest that maternal age and parity selectively interfere with ICOS expression in peripheral blood. In the context of the well‐established association between increasing parity and acquired immunity to PM [5], this pattern may reflect efficient pregnancy‐acquired immune memory, limiting antigen persistence and reducing the requirement for sustained cTfh activation. Indeed, higher percentages of activated malaria‐specific CD4^+^ T cells expressing ICOS have previously been reported in primigravidae women than in multigravidae women [75]. While this study implicates cTfh cells in immune responses to *P. falciparum* malaria during pregnancy, several limitations should be acknowledged. First, the statistical power needed to confirm certain associations was limited by the sample size, which was rather small. Second, there was no functional evaluation of cTfh activity, such as cytokine release and antibody induction, which restricts the ability to interpret the reported phenotypic variations mechanistically. Finally, the lack of data on the contribution of infection chronicity to cTfh cell activation due to the low sample size represents an important limitation of this study.

## 5. Conclusion

Overall, this study reveals a key role of cTfh cells in host immune responses during *P. falciparum* PM and pregnancy outcome. Therefore, understanding cTfh cell function and mechanisms during pregnancy may help facilitate the development of new therapeutic interventions targeting human PM.

**Nomenclature**
BTLA: B and T lymphocyte attenuator
CBMC: Cord blood mononuclear cells
CD4: Cluster of differentiation 4
cTfh cells: Circulating T follicular helper cells
ICOS: Inducible T cell co‐stimulation
IL: Interleukin
IVBMC: Intervillous blood mononuclear cells
LAG‐3: *Lymphocyte-activation gene 3*
PBMC: Peripheral blood mononuclear cells
PM+: Placental malaria positive
PM−: Placental malaria negative.

## Author Contributions

Rosette Megnekou, Bernard Marie Zambo Bitye, and Chris Marco Mbianda Nana conceived and designed the study. Bernard Marie Zambo Bitye, Chris Marco Mbianda Nana, Balotin Fogang, Bodin Darcisse Kwanou Tchakounte, Berenice Kenfack Tekougang Zangue, and Reine Seumko’o Medouen Ndeumou help with data acquisition. Bernard Marie Zambo Bitye, Chris Marco Mbianda Nana, and Bodin Darcisse Kwanou Tchakounte performed most of the experiments. Bernard Marie Zambo Bitye, Chris Marco Mbianda Nana, and Rosette Megnekou analyzed and interpreted the data. Bernard Marie Zambo Bitye, Chris Marco Mbianda Nana, and Rosette Megnekou drafted the manuscript. Rosette Megnekou and Lawrence Ayong substantively reviewed the final manuscript for important intellectual content. Rosette Megnekou supervised the study.

## Funding

This work was financially supported by the TWAS and SIDA research grant (Grant 20‐313 RG/BIO/AF/AC_G FR3240314171).

## Disclosure

The funder had no role in study design, data collection and analyses, decision to publish or preparation of the manuscript. All authors contributed to the article and approved the submitted manuscript.

## Ethics Statement

This study involving humans was approved by the National Ethics Committee of Cameroon (Ethical Clearances Number 2021/02/1331/CE/CNERSH/SP). Administrative authorizations were obtained from the Cameroon Ministry of Public Health (Number 631‐1221 of 28th April 2021, D30‐459/L/MINSANTE/SG/DROS). The studies were conducted in accordance with the local legislation and institutional requirements. Written informed consent for participation in this study was provided by the participants’ legal guardians/next of kin.

## Consent

The manuscript does not contain any individual person’s data.

## Conflicts of Interest

The authors declare no conflicts of interest.

## Supporting Information

Additional supporting information can be found online in the Supporting Information section.

## Supporting information


**Supporting Information 1** Table S1: Correlation between the frequency of cTfh cells in PBMC, IVBMC, and CBMC and pregnancy outcome. Spearman’s rank order correlation test was used to determine the relationship between the frequencies of cTfh cells and their subtypes in different blood compartments (PBMC, IVBMC, and CBMC) and pregnancy outcomes (maternal hemoglobin levels, birth weight, and fetal hemoglobin levels). Significant differences are shown in the graph. (PBMC: *n* = 49, IVBMC: *n* = 45, and CBMC: *n* = 48). CBMC, cord blood mononuclear cell; cTfh, circulating T follicular helper; geoMFI, geometric mean fluorescence intensity; IVBMC, intervillous blood mononuclear cell; PBMC, peripheral blood mononuclear cell; *r*_s_, Spearman rank correlation coefficient.

**Supporting Information 2** Table S2. Correlation between the frequency of cTfh cells in PBMC, IVBMC and CBMC, and pregnancy parameters. Spearman’s rank order correlation test was used to determine the relationship between the frequencies of cTfh cells and their subtypes in different blood compartments (PBMC, IVBMC, and CBMC) and pregnancy parameters (maternal age, gestational age, gravidity, and parity). Significant differences are shown in the graph. (PBMC: *n* = 49, IVBMC: *n* = 45, and CBMC: *n* = 48). CBMC, Cord blood mononuclear cell; cTfh, Circulating T follicular helper; geoMFI, Geometric mean fluorescence intensity; IVBMC, Intervillous blood mononuclear cell; PBMC, Peripheral blood mononuclear cell; *r*_s_, Spearman rank correlation coefficient.

## Data Availability

The original contributions presented in the study are included in the article. Further inquiries can be directed to the corresponding author.

## References

[1] World Malaria Report 2023 , Retrieved September 16, 2024 https://www.who.int/teams/global-malaria-programme/reports/world-malaria-report-2023.

[2] Guyatt H. L. and Snow R. W. , Impact of Malaria During Pregnancy on Low Birth Weight in Sub-Saharan Africa, Clinical Microbiology Reviews. (2004) 17, no. 4, 760–769, 10.1128/CMR.17.4.760-769.2004, 2-s2.0-5644251581. 15489346 PMC523568

[3] Megnekou R. , Djontu J. C. , Bigoga J. D. , Lissom A. , and Magagoum S. H. , Role of Some Biomarkers in Placental Malaria in Women Living in Yaoundé, Cameroon, Acta Tropica. (2015) 141, no. Pt A, 97–102, 10.1016/j.actatropica.2014.10.007, 2-s2.0-84910024263. 25447267

[4] Fried M. and Duffy P. E. , Adherence of *Plasmodium falciparum* to Chondroitin Sulfate A in the Human Placenta, Science. (1996) 272, no. 5267, 1502–1504, 10.1126/science.272.5267.1502, 2-s2.0-0029992826. 8633247

[5] Rogerson S. J. , Hviid L. , Duffy P. E. , Leke R. F. G. , and Taylor D. W. , Malaria in Pregnancy: Pathogenesis and Immunity, The Lancet Infectious Diseases. (2007) 7, no. 2, 105–117, 10.1016/S1473-3099(07)70022-1, 2-s2.0-33846315783. 17251081

[6] Salanti A. , Dahlbäck M. , and Turner L. , et al.Evidence for the Involvement of VAR2CSA in Pregnancy-Associated Malaria, The Journal of Experimental Medicine. (2004) 200, no. 9, 1197–1203, 10.1084/jem.20041579, 2-s2.0-8644292712. 15520249 PMC2211857

[7] Kumar R. , Loughland J. R. , Ng S. S. , Boyle M. J. , and Engwerda C. R. , The Regulation of CD4^+^ T Cells During Malaria, Immunological Reviews. (2020) 293, no. 1, 70–87, 10.1111/imr.12804. 31674682

[8] McLean A. R. D. , Opi D. H. , and Stanisic D. I. , et al.High Antibodies to VAR2CSA in Response to Malaria Infection Are Associated With Improved Birthweight in a Longitudinal Study of Pregnant Women, Frontiers in Immunology. (2021) 12, 10.3389/fimmu.2021.644563, 644563. 34220804 PMC8242957

[9] Crotty S. , T Follicular Helper Cell Biology: A Decade of Discovery and Diseases, Immunity. (2019) 50, no. 5, 1132–1148, 10.1016/j.immuni.2019.04.011, 2-s2.0-85065517725. 31117010 PMC6532429

[10] Brenna E. , Davydov A. N. , and Ladell K. , et al.CD4+ T Follicular Helper Cells in Human Tonsils and Blood Are Clonally Convergent but Divergent From Non-Tfh CD4+ Cells, Cell Reports. (2020) 30, no. 1, 137–152.e5, 10.1016/j.celrep.2019.12.016. 31914381 PMC7029615

[11] Hale J. S. and Ahmed R. , Memory T Follicular Helper CD4 T Cells, Frontiers in Immunology. (2015) 6, 10.3389/fimmu.2015.00016, 2-s2.0-84926645867, 16. 25699040 PMC4313784

[12] Morita R. , Schmitt N. , and Bentebibel S.-E. , et al.Human Blood CXCR5(+)CD4(+) T Cells Are Counterparts of T Follicular Cells and Contain Specific Subsets That Differentially Support Antibody Secretion, Immunity. (2011) 34, no. 1, 108–121, 10.1016/j.immuni.2010.12.012, 2-s2.0-78751690647. 21215658 PMC3046815

[13] Schmitt N. , Bentebibel S.-E. , and Ueno H. , Phenotype and Functions of Memory Tfh Cells in Human Blood, Trends in Immunology. (2014) 35, no. 9, 436–442, 10.1016/j.it.2014.06.002, 2-s2.0-84922551023. 24998903 PMC4152409

[14] Fu Y. , Li L. , and Liu X. , et al.Estrogen Promotes B Cell Activation In Vitro Through Down-Regulating CD80 Molecule Expression, Gynecological Endocrinology. (2011) 27, no. 8, 593–596, 10.3109/09513590.2010.507281, 2-s2.0-79959937309. 21726119

[15] Kay A. W. , Bayless N. L. , and Fukuyama J. , et al.Pregnancy Does Not Attenuate the Antibody or Plasmablast Response to Inactivated Influenza Vaccine, Journal of Infectious Diseases. (2015) 212, no. 6, 861–870, 10.1093/infdis/jiv138, 2-s2.0-84935098231. 25740957 PMC4548461

[16] Robinson D. P. and Klein S. L. , Pregnancy and Pregnancy-Associated Hormones Alter Immune Responses and Disease Pathogenesis, Hormones and Behavior. (2012) 62, no. 3, 263–271, 10.1016/j.yhbeh.2012.02.023, 2-s2.0-84866759978. 22406114 PMC3376705

[17] Chan J.-A. , Loughland J. R. , and de Labastida Rivera F. , et al.Th2-Like T Follicular Helper Cells Promote Functional Antibody Production During, *Plasmodium falciparum*, Infection, Cell Reports Medicine. (2020) 1, no. 9, 10.1016/j.xcrm.2020.100157, 100157. 33377128 PMC7762767

[18] Oyong D. A. , Loughland J. R. , and Soon M. S. F. , et al.Adults With Plasmodium Falciparum Malaria Have Higher Magnitude and Quality of Circulating T-Follicular Helper Cells Compared to Children, EBioMedicine. (2022) 75, 10.1016/j.ebiom.2021.103784, 103784. 34968760 PMC8718734

[19] Monteiro C. , Kasahara T. , and Castro J. , et al.Pregnancy Favors the Expansion of Circulating Functional Follicular Helper T Cells, Journal of Reproductive Immunology. (2017) 121, 1–10, 10.1016/j.jri.2017.04.007, 2-s2.0-85018735175. 28482188

[20] Zeng W. , Liu Z. , and Zhang S. , et al.Characterization of T Follicular Helper Cells in Allogeneic Normal Pregnancy and PDL1 Blockage-Induced Abortion, Scientific Reports. (2016) 6, no. 1, 10.1038/srep36560, 2-s2.0-84994545639, 36560. 27819282 PMC5098204

[21] Chen L. and Flies D. B. , Molecular Mechanisms of T Cell Co-Stimulation and Co-Inhibition, Nature Reviews Immunology. (2013) 13, no. 4, 227–242, 10.1038/nri3405, 2-s2.0-84875463042. PMC3786574 23470321

[22] Hurchla M. A. , Sedy J. R. , Gavrieli M. , Drake C. G. , Murphy T. L. , and Murphy K. M. , B and T Lymphocyte Attenuator Exhibits Structural and Expression Polymorphisms and Is Highly Induced in Anergic CD4+ T Cells, The Journal of Immunology. (2005) 174, no. 6, 3377–3385, 10.4049/jimmunol.174.6.3377, 2-s2.0-14844357247. 15749870

[23] Watanabe N. , Gavrieli M. , and Sedy J. R. , et al.BTLA Is a Lymphocyte Inhibitory Receptor With Similarities to CTLA-4 and PD-1, Nature Immunology. (2003) 4, no. 7, 670–679, 10.1038/ni944, 2-s2.0-0037810670. 12796776

[24] Sedy J. R. , Gavrieli M. , and Potter K. G. , et al.B and T Lymphocyte Attenuator Regulates T Cell Activation Through Interaction With Herpesvirus Entry Mediator, Nature Immunology. (2005) 6, no. 1, 90–98, 10.1038/ni1144, 2-s2.0-19944433635. 15568026

[25] Chtanova T. , Tangye S. G. , and Newton R. , et al.T Follicular Helper Cells Express a Distinctive Transcriptional Profile, Reflecting Their Role as Non-Th1/Th2 Effector Cells That Provide Help for B Cells, The Journal of Immunology. (2004) 173, no. 1, 68–78, 10.4049/jimmunol.173.1.68, 2-s2.0-2942755979. 15210760

[26] Kashiwakuma D. , Suto A. , and Hiramatsu Y. , et al.B and T Lymphocyte Attenuator Suppresses IL-21 Production From Follicular Th Cells and Subsequent Humoral Immune Responses, The Journal of Immunology. (2010) 185, no. 5, 2730–2736, 10.4049/jimmunol.0903839, 2-s2.0-78049354525. 20660710

[27] Triebel F. , Jitsukawa S. , and Baixeras E. , et al.LAG-3, a Novel Lymphocyte Activation Gene Closely Related to CD4, The Journal of Experimental Medicine. (1990) 171, no. 5, 1393–1405, 10.1084/jem.171.5.1393, 2-s2.0-0025338920. 1692078 PMC2187904

[28] Herrmann M. , Schulte S. , and Wildner N. H. , et al.Analysis of Co-Inhibitory Receptor Expression in COVID-19 Infection Compared to Acute Plasmodium Falciparum Malaria: LAG-3 and TIM-3 Correlate With T Cell Activation and Course of Disease, Frontiers in Immunology. (2020) 11, 10.3389/fimmu.2020.01870, 1870. 32983106 PMC7479337

[29] Illingworth J. , Butler N. S. , and Roetynck S. , et al.Chronic Exposure to *Plasmodium falciparum* Is Associated With Phenotypic Evidence of B and T Cell Exhaustion, The Journal of Immunology. (2013) 190, no. 3, 1038–1047, 10.4049/jimmunol.1202438. 23264654 PMC3549224

[30] Legat A. , Speiser D. E. , Pircher H. , Zehn D. , and Fuertes Marraco S. A. , Inhibitory Receptor Expression Depends More Dominantly on Differentiation and Activation Than “Exhaustion” of Human CD8 T Cells, Frontiers in Immunology. (2013) 4, 10.3389/fimmu.2013.00455, 2-s2.0-84892146573, 455. 24391639 PMC3867683

[31] Abel A. , Steeg C. , and Aminkiah F. , et al.Differential Expression Pattern of Co-Inhibitory Molecules on CD4+ T Cells in Uncomplicated Versus Complicated Malaria, Scientific Reports. (2018) 8, no. 1, 10.1038/s41598-018-22659-1, 2-s2.0-85044248387, 4789. 29555909 PMC5859076

[32] Costa P. A. C. , Leoratti F. M. S. , and Figueiredo M. M. , et al.Induction of Inhibitory Receptors on T Cells During *Plasmodium vivax* Malaria Impairs Cytokine Production, Journal of Infectious Diseases. (2015) 212, no. 12, 1999–2010, 10.1093/infdis/jiv306, 2-s2.0-84959440940. 26019284 PMC4655853

[33] Jacobs T. , Plate T. , Gaworski I. , and Fleischer B. , CTLA-4-Dependent Mechanisms Prevent T Cell Induced-Liver Pathology During the Erythrocyte Stage of *Plasmodium berghei* Malaria, European Journal of Immunology. (2004) 34, no. 4, 972–980, 10.1002/eji.200324477, 2-s2.0-3843133171. 15048707

[34] Wykes M. N. , Horne-Debets J. M. , Leow C.-Y. , and Karunarathne D. S. , Malaria Drives T Cells to Exhaustion, Frontiers in Microbiology. (2014) 5, 10.3389/fmicb.2014.00249, 2-s2.0-84904884650, 249. 24904561 PMC4034037

[35] World Health Organization (WHO) , Global Nutrition Targets 2025: Low Birth Weight Policy Brief, n.d., Retrieved September 25, 2024 https://www.who.int/publications/i/item/WHO-NMH-NHD-14.5.

[36] World Health Organization (WHO) , Universal Access to Malaria Diagnostic Testing: An Operational Manual, 2011.

[37] Bulmer J. N. , Rasheed F. N. , Francis N. , Morrison L. , and Greenwood B. M. , Placental Malaria. I. Pathological Classification, Histopathology. (1993) 22, no. 3, 211–218, 10.1111/j.1365-2559.1993.tb00110.x, 2-s2.0-0027404930. 8495954

[38] Fried M. , Muehlenbachs A. , and Duffy P. E. , Diagnosing Malaria in Pregnancy: An Update, Expert Review of Anti-Infective Therapy. (2012) 10, no. 10, 1177–1187, 10.1586/eri.12.98, 2-s2.0-84870597540. 23199403 PMC3552641

[39] Polin R. A. , Fox W. W. , and Abman S. H. , Fetal and Neonatal Physiology, 2011, 4th editionn, Elsevier/Saunders.

[40] Nana C. M. M. , Tchakounté B. D. K. , and Bitye B. M. Z. , et al.Phenotypic Changes of γδ T Cells in Plasmodium Falciparum Placental Malaria and Pregnancy Outcomes in Women at Delivery in Cameroon, Frontiers in Immunology. (2024) 15, 10.3389/fimmu.2024.1385380, 1385380. 38827744 PMC11140112

[41] Locci M. , Havenar-Daughton C. , and Landais E. , et al.Human Circulating PD-1+CXCR3−CXCR5+ Memory Tfh Cells Are Highly Functional and Correlate With Broadly Neutralizing HIV Antibody Responses, Immunity. (2013) 39, no. 4, 758–769, 10.1016/j.immuni.2013.08.031, 2-s2.0-84885743368. 24035365 PMC3996844

[42] Cárdeno A. , Magnusson M. K. , Quiding-Järbrink M. , and Lundgren A. , Activated T Follicular Helper-Like Cells Are Released Into Blood After Oral Vaccination and Correlate With Vaccine Specific Mucosal B-Cell Memory, Scientific Reports. (2018) 8, no. 1, 10.1038/s41598-018-20740-3, 2-s2.0-85053630301, 2729. 29426881 PMC5807513

[43] Herati R. S. , Silva L. V. , and Vella L. A. , et al.Vaccine-Induced ICOS+CD38+ Circulating Tfh Are Sensitive Biosensors of Age-Related Changes in Inflammatory Pathways, Cell Reports Medicine. (2021) 2, no. 5, 10.1016/j.xcrm.2021.100262, 100262. 34095875 PMC8149371

[44] Soon M. S. F. , Nalubega M. , and Boyle M. J. , T-Follicular Helper Cells in Malaria Infection and Roles in Antibody Induction, Oxford Open Immunology. (2021) 2, no. 1, 10.1093/oxfimm/iqab008, iqab008. 36845571 PMC9914587

[45] Luan X. , Kang X. , Li W. , and Dong Q. , An Investigation of the Relationship Between Recurrent Spontaneous Abortion and Memory T Follicular Helper Cells, American Journal of Reproductive Immunology. (2017) 78, no. 5, 10.1111/aji.12714, 2-s2.0-85021253173, e12714. 28639391

[46] Okamgba O. C. , Ifeanyichukwu M. O. , Ilesanmi A. O. , and Chigbu L. N. , Variations in the Leukocyte and Cytokine Profiles Between Placental and Maternal Circulation in Pregnancy-Associated Malaria, Research and Reports in Tropical Medicine. (2018) 9, 1–8, 10.2147/RRTM.S137829. 30050350 PMC6047617

[47] Ma C. S. , Suryani S. , and Avery D. T. , et al.Early Commitment of Naïve Human CD4^+^ T Cells to the T Follicular Helper (T_FH_) Cell Lineage is Induced by IL-12, Immunology & Cell Biology. (2009) 87, no. 8, 590–600, 10.1038/icb.2009.64, 2-s2.0-70450265288. 19721453

[48] Heydarlou H. , Eghabl-Fard S. , and Ahmadi M. , et al.Investigation of Follicular Helper T Cells, as a Novel Player, in Preeclampsia, Journal of Cellular Biochemistry. (2019) 120, no. 3, 3845–3852, 10.1002/jcb.27666, 2-s2.0-85053925830. 30259994

[49] Chan J.-A. , Loughland J. R. , and De La Parte L. , et al.Age-Dependent Changes in Circulating Tfh Cells Influence Development of Functional Malaria Antibodies in Children, Nature Communications. (2022) 13, no. 1, 10.1038/s41467-022-31880-6, 4159. PMC9293980 35851033

[50] Djontu J. C. , Siewe Siewe S. , and Mpeke Edene Y. D. , et al.Impact of Placental Plasmodium Falciparum Malaria Infection on the Cameroonian Maternal and Neonate’s Plasma Levels of Some Cytokines Known to Regulate T Cells Differentiation and Function, Malaria Journal. (2016) 15, no. 1, 10.1186/s12936-016-1611-0, 2-s2.0-84995743412, 561. 27871325 PMC5117507

[51] Fried M. , Kurtis J. D. , and Swihart B. , et al.Systemic Inflammatory Response to Malaria During Pregnancy Is Associated With Pregnancy Loss and Preterm Delivery, Clinical Infectious Diseases. (2017) 65, no. 10, 1729–1735, 10.1093/cid/cix623, 2-s2.0-85032731653. 29020221 PMC5850414

[52] Omer S. , Franco-Jarava C. , and Noureldien A. , et al.Impact of Placental Malaria on Maternal, Placental and Fetal Cord Responses and Its Role in Pregnancy Outcomes in Women From Blue Nile State, Sudan, Malaria Journal. (2021) 20, no. 1, 10.1186/s12936-021-03580-x, 35. 33422078 PMC7797158

[53] Currenti J. , Simmons J. , and Oakes J. , et al.Tracking of Activated cTfh Cells Following Sequential Influenza Vaccinations Reveals Transcriptional Profile of Clonotypes Driving a Vaccine-Induced Immune Response, Frontiers in Immunology. (2023) 14, 10.3389/fimmu.2023.1133781, 1133781. 37063867 PMC10095155

[54] Huber J. E. , Ahlfeld J. , and Scheck M. K. , et al.Dynamic Changes in Circulating T Follicular Helper Cell Composition Predict Neutralising Antibody Responses After Yellow Fever Vaccination, Clinical & Translational Immunology. (2020) 9, no. 5, 10.1002/cti2.1129, e1129. 32419947 PMC7221214

[55] Lu X. , Zhang X. , and Cheung A. K. L. , et al.Abnormal Shift in B Memory Cell Profile Is Associated With the Expansion of Circulating T Follicular Helper Cells via ICOS Signaling During Acute HIV-1 Infection, Frontiers in Immunology. (2022) 13, 10.3389/fimmu.2022.837921, 837921. 35222430 PMC8867039

[56] Zhang Y. , Jiang Y. , and Wang Y. , et al.Higher Frequency of Circulating PD-1^high^ CXCR5^+^ CD4^+^ Tfh Cells in Patients With Chronic Schistosomiasis, International Journal of Biological Sciences. (2015) 11, no. 9, 1049–1055, 10.7150/ijbs.12023, 2-s2.0-84937685640. 26221072 PMC4515816

[57] Hutloff A. , Dittrich A. M. , and Beier K. C. , et al.ICOS Is an Inducible T-Cell Co-Stimulator Structurally and Functionally Related to CD28, Nature. (1999) 397, no. 6716, 263–266, 10.1038/16717, 2-s2.0-0033590502. 9930702

[58] Mintz M. A. and Cyster J. G. , T Follicular Helper Cells in Germinal Center B Cell Selection and Lymphomagenesis, Immunological Reviews. (2020) 296, no. 1, 48–61, 10.1111/imr.12860. 32412663 PMC7817257

[59] Vinuesa C. G. , Linterman M. A. , Yu D. , and MacLennan I. C. M. , Follicular Helper T Cells, Annual Review of Immunology. (2016) 34, no. 1, 335–368, 10.1146/annurev-immunol-041015-055605, 2-s2.0-84969759563. 26907215

[60] Rasheed A.-U. , Rahn H.-P. , Sallusto F. , Lipp M. , and Müller G. , Follicular B Helper T Cell Activity is Confined to CXCR5 ^hi^ ICOS ^hi^ CD4 T Cells and is Independent of CD57 Expression, European Journal of Immunology. (2006) 36, no. 7, 1892–1903, 10.1002/eji.200636136, 2-s2.0-33746256850. 16791882

[61] Steinberg M. W. , Cheung T. C. , and Ware C. F. , The Signaling Networks of the Herpesvirus Entry Mediator (TNFRSF14) in Immune Regulation, Immunological Reviews. (2011) 244, no. 1, 169–187, 10.1111/j.1600-065X.2011.01064.x, 2-s2.0-80054850189. 22017438 PMC3381650

[62] Larsson M. , Shankar E. M. , and Che K. F. , et al.Molecular Signatures of T-Cell Inhibition in HIV-1 Infection, Retrovirology. (2013) 10, no. 1, 10.1186/1742-4690-10-31, 2-s2.0-84875078589, 31. 23514593 PMC3610157

[63] Watanabe N. and Nakajima H. , Coinhibitory Molecules in Autoimmune Diseases, Clinical and Developmental Immunology. (2012) 2012, 10.1155/2012/269756, 2-s2.0-84867379035, 269756. 22997525 PMC3446788

[64] Karabon L. , Partyka A. , and Ciszak L. , et al.Abnormal Expression of BTLA and CTLA-4 Immune Checkpoint Molecules in Chronic Lymphocytic Leukemia Patients, Journal of Immunology Research. (2020) 2020, no. 1, 10.1155/2020/6545921, 6545921. 32775467 PMC7407019

[65] Otsuki N. , Kamimura Y. , Hashiguchi M. , and Azuma M. , Expression and Function of the B and T Lymphocyte Attenuator (BTLA/CD272) on Human T Cells, Biochemical and Biophysical Research Communications. (2006) 344, no. 4, 1121–1127, 10.1016/j.bbrc.2006.03.242, 2-s2.0-33646171259. 16643847

[66] Ahmadivand S. , Fux R. , and Palić D. , Role of T Follicular Helper Cells in Viral Infections and Vaccine Design, Cells. (2025) 14, no. 7, 10.3390/cells14070508, 508. 40214462 PMC11987902

[67] Vella L. A. , Herati R. S. , and Wherry E. J. , CD4+ T Cell Differentiation in Chronic Viral Infections: The Tfh Perspective, Trends in Molecular Medicine. (2017) 23, no. 12, 1072–1087, 10.1016/j.molmed.2017.10.001, 2-s2.0-85033562709. 29137933 PMC5886740

[68] Solders M. , Gorchs L. , Gidlöf S. , Tiblad E. , Lundell A.-C. , and Kaipe H. , Maternal Adaptive Immune Cells in Decidua Parietalis Display a More Activated and Coinhibitory Phenotype Compared to Decidua Basalis, Stem Cells International. (2017) 2017, 10.1155/2017/8010961, 2-s2.0-85042863985, 8010961. 29317870 PMC5727765

[69] Burton G. J. and Jauniaux E. , Pathophysiology of Placental-Derived Fetal Growth Restriction, American Journal of Obstetrics and Gynecology. (2018) 218, no. 2, S745–S761, 10.1016/j.ajog.2017.11.577, 2-s2.0-85044663418. 29422210

[70] Hu X.-H. , Tang M.-X. , Mor G. , and Liao A.-H. , Tim-3: Expression on Immune Cells and Roles at the Maternal-Fetal Interface, Journal of Reproductive Immunology. (2016) 118, 92–99, 10.1016/j.jri.2016.10.113, 2-s2.0-84992477566. 27792886

[71] Suguitan A. L. , Leke R. G. F. , and Fouda G. , et al.Changes in the Levels of Chemokines and Cytokines in the Placentas of Women With *Plasmodium falciparum* Malaria, The Journal of Infectious Diseases. (2003) 188, no. 7, 1074–7082, 10.1086/378500, 2-s2.0-0142105949. 14513430

[72] Boudová S. , Divala T. , Mungwira R. , Mawindo P. , Tomoka T. , and Laufer M. K. , Placental but Not Peripheral Plasmodium Falciparum Infection During Pregnancy Is Associated With Increased Risk of Malaria in Infancy, The Journal of Infectious Diseases. (2017) 216, no. 6, 732–735, 10.1093/infdis/jix372, 2-s2.0-85030610856. 28934438 PMC5853669

[73] Malhotra I. , Dent A. , and Mungai P. , et al.Can Prenatal Malaria Exposure Produce an Immune Tolerant Phenotype? A Prospective Birth Cohort Study in Kenya, PLoS Medicine. (2009) 6, no. 7, 10.1371/journal.pmed.1000116, 2-s2.0-68049144807, e1000116. 19636353 PMC2707618

[74] Mutabingwa T. K. , Bolla M. C. , and Li J.-L. , et al.Maternal Malaria and Gravidity Interact to Modify Infant Susceptibility to Malaria, PLoS Medicine. (2005) 2, no. 12, 10.1371/journal.pmed.0020407, 2-s2.0-29644438828, e407. 16259531 PMC1277932

[75] Kirosingh A. S. , Delmastro A. , and Kakuru A. , et al.Malaria-Specific Type 1 Regulatory T Cells Are More Abundant In First Pregnancies and Associated With Placental Malaria, eBioMedicine. (2023) 95, 10.1016/j.ebiom.2023.104772, 104772. 37634385 PMC10474374

