# Supplementary material for: cTfh Cell Activation and Changes in BTLA and LAG‐3 Expression Differentially Associate With Poor Pregnancy Outcome in Plasmodium falciparum Placental Malaria
Source: J Immunol Res. 2026 Apr 15;2026:6467951. doi: 10.1155/jimr/6467951 (PMC13581053; doi:10.1155/jimr/6467951)
Supplement: Supplementary file 2 [file JIMR-2026-6467951-s002.docx]

**Supplementary Table 2: Correlation between the frequency of cTfh cells in PBMC, IVBMC and CBMC and pregnancy parameters.**

**B**

**C**

**A**

Spemann's rank order correlation test was used to determine the relationship between the frequencies of cTfh cells and their subtypes in different blood compartments (PBMC, IVBMC, and CBMC) and pregnancy parameters (maternal age, gestational age, gravidity and parity). **r_s_**: Spemann Rank correlation coefficient; **cTfh**: Circulating T follicular helper; **geoMFI**: geometric mean fluorescence intensity; **PBMC**: Peripheral blood mononuclear cell; **IVBMC**: Intervillous blood mononuclear cell; **CBMC**: Cord blood mononuclear cell.
